# Using a combination therapy to combat scalp necrosis: a case report

**DOI:** 10.1186/s13256-020-02450-5

**Published:** 2020-08-20

**Authors:** Kazem Hajmohammadi, Roghayeh Esmaeili Zabihi, Kamran Akbarzadeh, Naser Parizad

**Affiliations:** 1 Imam Khomeini Teaching Hospital, Urmia University of Medical Sciences, Imam Khomeini Hospital, Ershad Ave., Urmia, 571478-3734 Iran; 2 Department of Nursing, Faculty of Nursing and Midwifery, Urmia University of Medical Sciences, Campus Nazlu, 11 KM Road Seru, Urmia, West Azerbaijan 575611-5111 Iran; 3 Department of Medical Entomology and Vector Control, School of Public Health, Tehran University of Medical Sciences, Enqelab Square, Tehran, 1417613151 Iran; 4 Patient Safety Research Center, Faculty of Nursing and Midwifery, Nursing and Midwifery Faculty, Campus Nazlu, 11 KM Road Seru, Urmia, West Azerbaijan 575611-5111 Iran

**Keywords:** Maggot debridement therapy, Negative-pressure wound therapy, Amniotic membrane grafting, Scalp, Necrosis, Diabetes mellitus, Iran

## Abstract

**Background:**

Chronic nonhealing wounds are very expensive to treat and debilitating, and they reduce health-related quality of life. Scalp necrosis is very rare due to its rich vascularity. However, any post-traumatic wounds with secondary infection can lead to scalp necrosis.

**Case presentation:**

We report a case of a 77-year-old Azerbaijani man with a history of diabetes who had a car accident and sustained a scalp wound. He underwent reconstructive surgery for the scalp wound. The wound became infected, and scalp necrosis developed following the surgery. There was no progress in wound healing in spite of conventional wound therapy. We combined maggot debridement therapy with negative-pressure wound therapy and amniotic membrane grafting for 7 months. Necrotic tissues began to be eliminated after the second use of larva therapy, and the wound became free of necrotic tissues with clear increase of granulated tissues after four treatments with maggot debridement therapy. Then, we applied negative-pressure wound therapy and amniotic membrane grafting to accelerate wound healing and improve wound closure. The patient’s scalp wound recovered well, and he was discharged to home in good condition.

**Conclusions:**

Medical and wound care teams can benefit from this combination therapy when dealing with nonhealing necrotic wounds.

## Background

Chronic nonhealing wounds are very expensive to treat, and they are a debilitating condition that reduces health-related quality of life [1]. Diabetes, obesity, medications, and aging are among multiple risk factors that rapidly increase the prevalence of chronic nonhealing wounds [1, 2]. Many chronic nonhealing wounds need alternative combination treatment in addition to conventional therapies [3]. Debridement is an essential factor to help wound healing [1]. One of the excellent forms of debridement is maggot debridement therapy (MDT), which helps in healing chronic wounds by facilitating debridement of necrotic tissue [4]. The effectiveness of MDT has been known since ancient times [5]. Its popularity declined with the discovery of antibiotics [1]. Because antimicrobial resistance has been rising in recent decades, MDT has become an increasingly popular and preferred method of treatment [6]. Negative-pressure wound therapy (NPWT) is another modern therapeutic technique that supports wound healing by increasing local blood flow, inhibiting bacterial growth, decreasing tissue edema, and eliminating exudates and proinflammatory cytokines [7]. The promising effect of amniotic membrane grafting (AMG) in treating chronic wounds was confirmed by recent studies [8, 9]. Application of modern wound care management in treating chronic nonhealing wounds has recently become very popular in Iran [10–12]. We present one case of a patient with a post-traumatic scalp wound who developed scalp necrosis following reconstructive surgery. He was successfully treated using MDT in combination with NPWT, AMG, silver-containing dressing, and antibiotic therapy.

## Case presentation

**Fig. 1:**
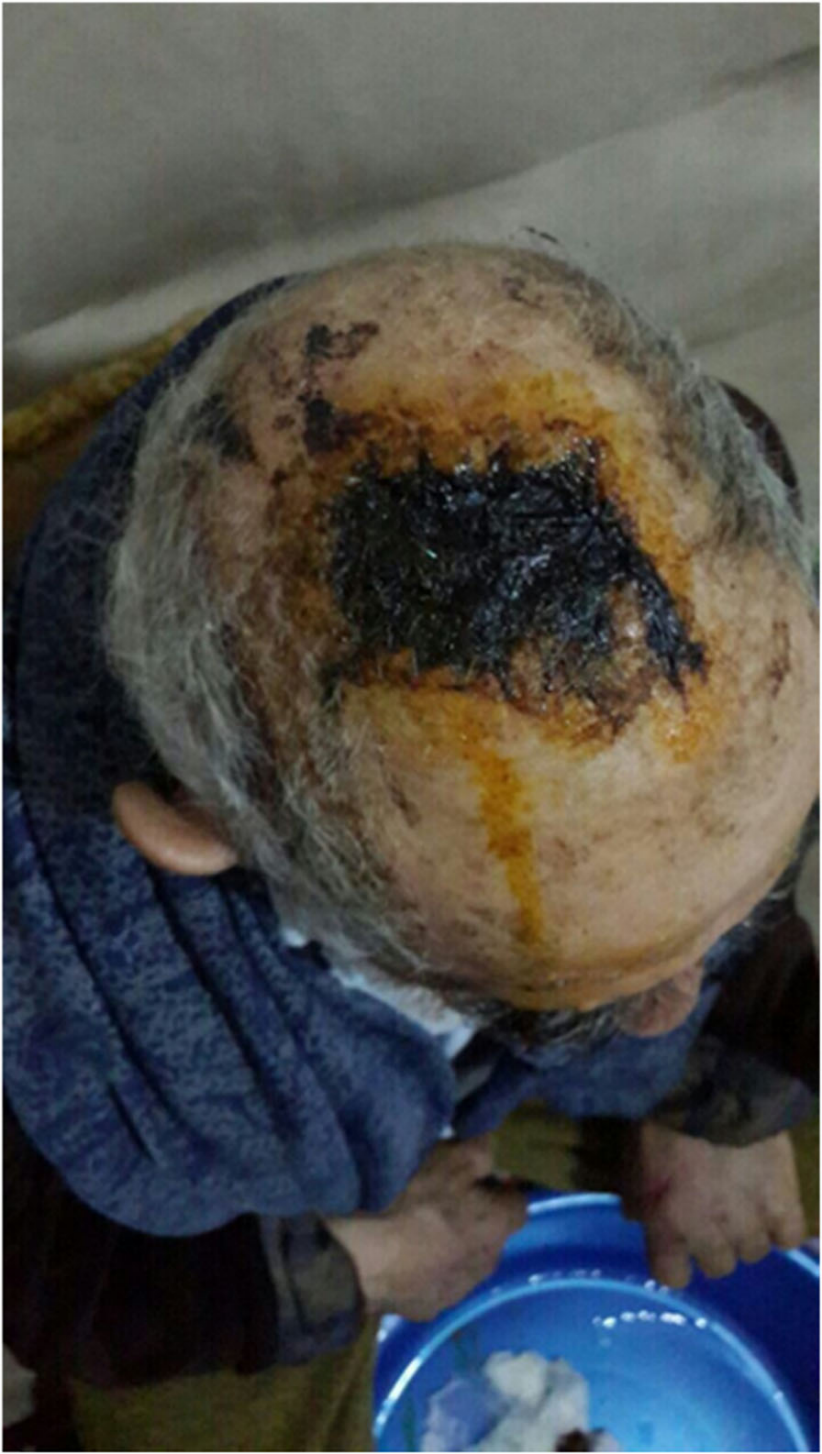
Extensive infected necrotic scalp wound before starting treatment

**Fig. 2:**
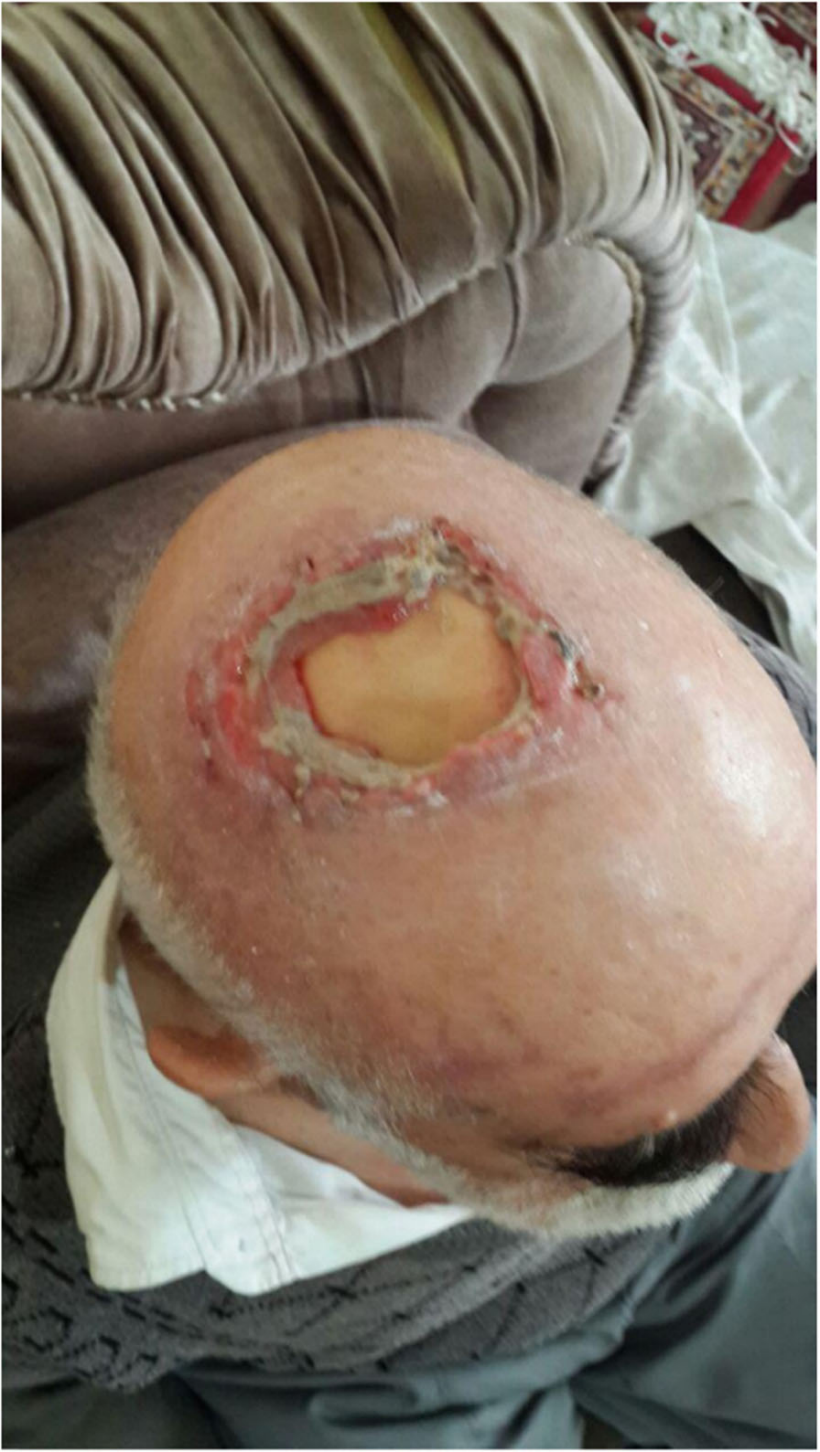
Exposed skull after debridement

**Fig. 3:**
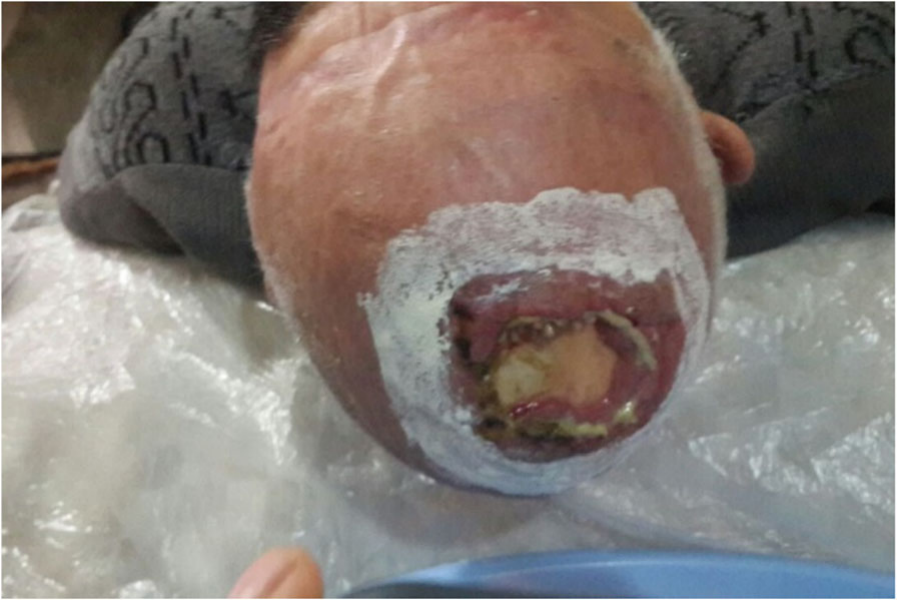
Maggots of *Lucilia sericata* in head scalp wound

**Fig. 4:**
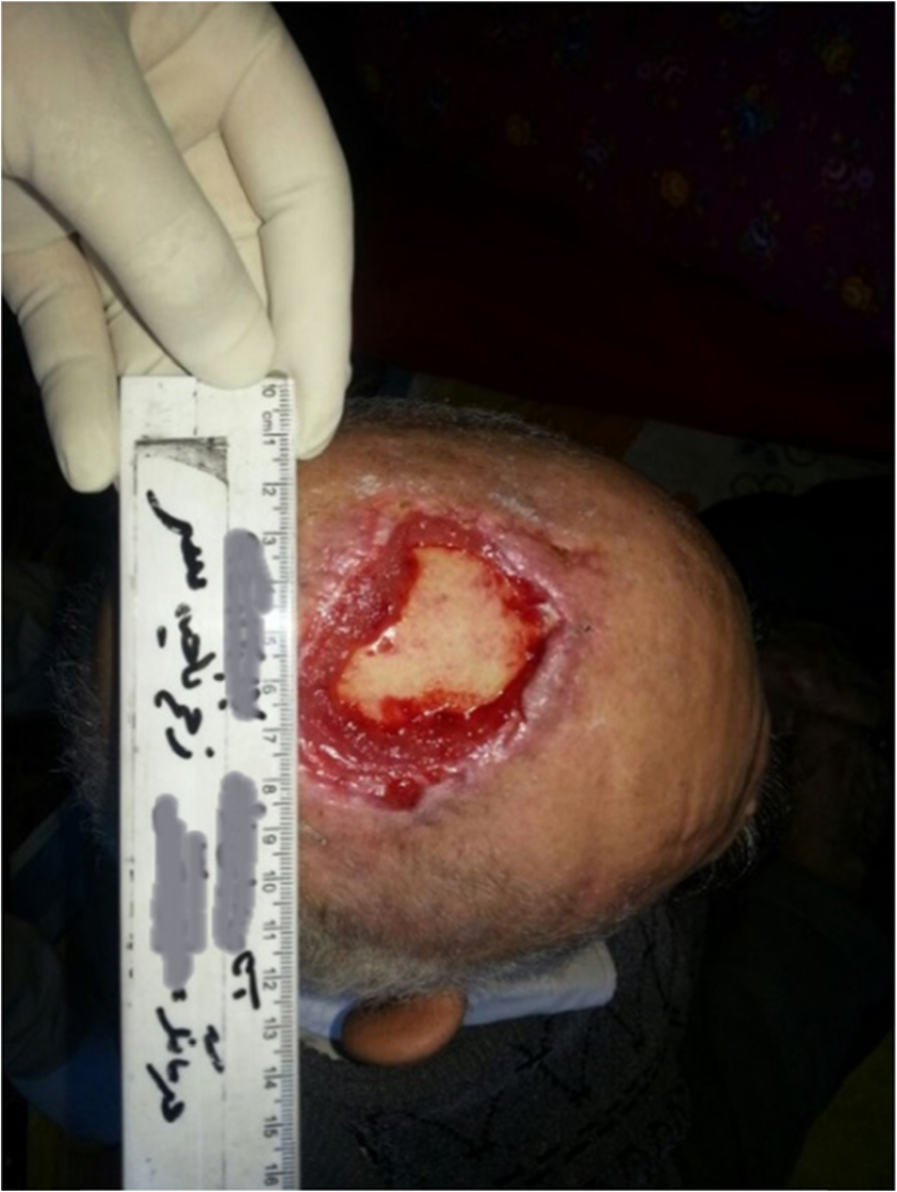
Wound status after four treatments of larval therapy during a 2-week period

**Fig. 5:**
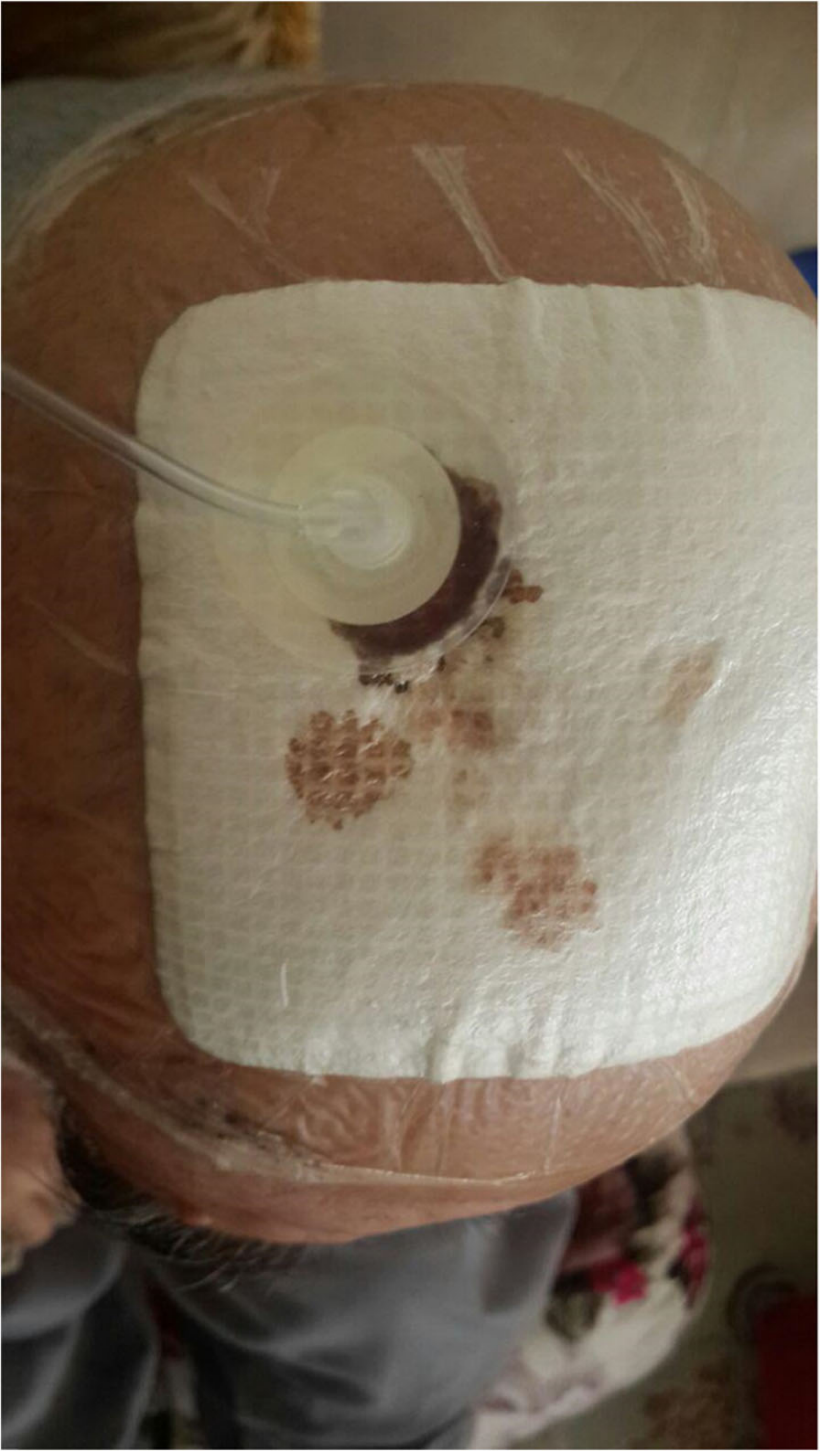
Negative-pressure wound therapy applied following maggot debridement therapy

**Fig. 6:**
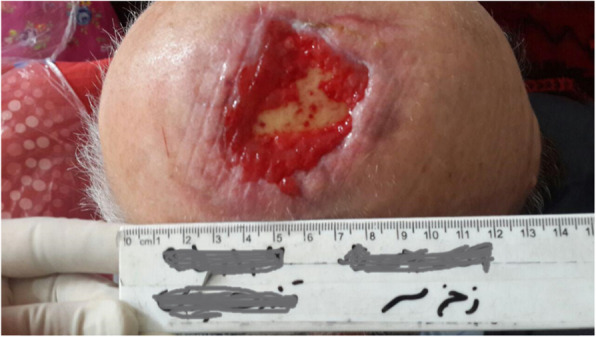
Reducing the exposed area by reepithelialization following amniotic membrane grafting

**Fig. 7:**
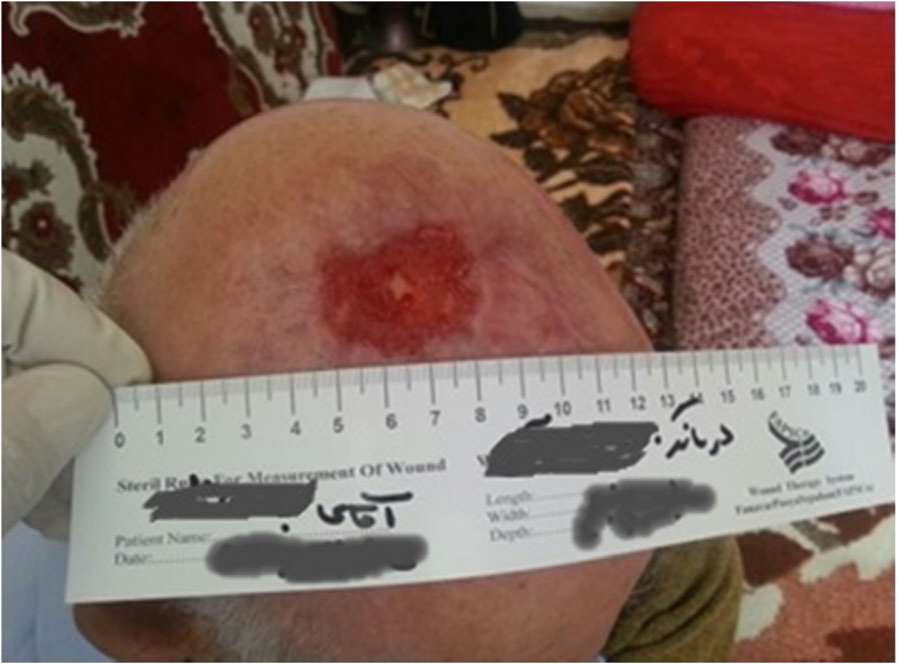
The final step of reducing the wound area and recovery of the head skin

**Fig. 8:**
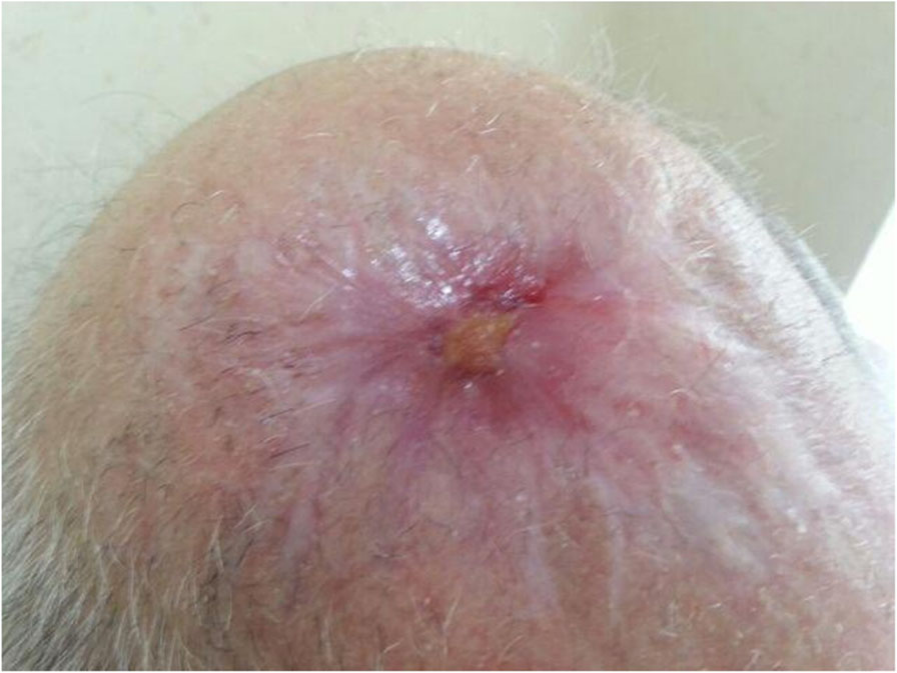
The patient was discharged to home after scalp wound healing

Our patient was a 77-year-old Azerbaijani man from Urmia, a city in northwestern Iran. He was admitted to our hospital following a scalp wound sustained in a car accident. The result of his initial clinical examination was normal, and his brain computed tomographic scan was negative for traumatic intracranial hemorrhage and skull fractures. Some of his lab results in admission were as follows: hemoglobin A1c 7.4%, blood sugar 265 mg/dl, high-density lipoprotein 35 mg/dl, low-density lipoprotein 71 mg/dl, cholesterol 142 mg/dl, and triglycerides 75 mg/dl. His medications included carvedilol 3.125 mg, Nitroglycerin 6.4 mg, aspirin 100 mg, Lantus insulin 10 units, and atorvastatin 40 mg. He had a history of type 2 diabetes, hypertension, and hyperlipidemia. He had atherosclerosis and had undergone angiography and stent implantation 12 years ago. He had a family history of diabetes, hypertension, and coronary artery disease. He is a smoker who smoked one pack per day. He denied addiction to any kind of drugs or alcohol. The patient was hospitalized with an extensive scalp wound and underwent reconstructive surgery for the scalp wound on April 20, 2019. The scalp wound became infected, and he developed scalp necrosis 4 days after the surgery (Fig. 1). The patient received saline wound irrigation and wet-to-dry dressing twice per day. He had no improvement despite receiving conventional treatments, and he was referred to the wound management team. In the first step, necrotic tissues were removed by autolytic debridement, and the skull was exposed (Fig. 2). Then, we used MDT for further debridement and disinfection of the wounds from bacterial infections as well as tending to increase granulated tissues on the wound. The edges of the wound were stimulated because of the larval secretion. This process helps maggots to immediately start their activity and increase their output. Sterile maggots for this case study were prepared in the Laboratory of Medicinal Flies in the Department of Medical Entomology, School of Public Health, Tehran University of Medical Sciences, Tehran, Iran. The maggots were L1 larvae of *Lucilia sericata* (Diptera: Calliphoridae). The maggot therapy procedure included preparation of the wound, release of the larvae on the wound, and dressing and removing of larvae after 48 hours. Next, larval therapy may be done immediately after removing third-instar larvae or after some time gaps. The peripheral area of the wound was prepared with zinc oxide to prevent irritation that may be produced by secretions of larvae (Fig. 3). After two treatments with larval therapy, all the necrotic tissues were totally removed, and granulation tissues appeared (Fig. 4). After four treatments with MDT in 3 weeks, silver-containing dressing and NPWT were applied to the wound for 7 months in order to make granulation tissue grow faster and promote the healing process (Fig. 5). AMG was used to accelerate epithelialization of the wound in the last month of treatment (Fig. 6). The scalp wound recovered well and totally closed (Fig. 7). The patient was discharged to home in good condition and is enjoying life with the healed wound (Fig. 8). No adverse effects were reported during or after therapeutic intervention.

## Discussion and conclusions

In this study, we report a case of an elderly patient with type 2 diabetes who had a car accident and sustained a scalp wound. This patient was at higher risk of developing wound infection and impaired wound healing due to diabetes and old age [1, 2]. Scalp wounds usually heal very well and rarely become necrotic because of their extensive vascularity. However, any post-traumatic wounds with secondary infection can lead to scalp necrosis [13]. Our patient’s scalp wound was infected and turned into necrotic tissue despite conventional therapies. Management of chronic wounds is challenging and requires other unconventional therapies in order to achieve healing [3]. We used MDT in combination with NPWT and AMG in order to achieve a better result in shorter time. Sun *et al.* showed that MDT shortened not only the time of healing but also the healing rate of chronic wounds [14]. MDT helps to improve the chronic wound with different mechanisms, such as with maggots that secrete proteolytic enzymes, which enable them to ingest necrotic tissue and degrade wound eschar [15]. The pH levels of a wound increase because of the maggot antibacterial secretions. This in turn enhances the healing process due to the elimination of bacteria [1]. Maggot secretions also enhance the formation of plasmin and induce fibrinolysis, resulting in the breakdown of the fibrin slough that accumulates in chronic wounds. This keeps the wound free of infection and inflammation to improve wound closure [16]. In line with our report, the positive impact of MDT on chronic nonhealing wounds has been confirmed in numerous studies [1, 3, 10, 15]. Futrega *et al.* recommended combination therapies to support effective and reliable wound treatment [17]. At this stage, we applied NPWT to enhance wound healing by increasing local blood flow, reducing tissue edema, eliminating exudates, promoting cell hyperplasia, and preventing bacterial growth [7]. Similar to our report, some recent studies have also confirmed that NPWT is a safe and effective technique to accelerate wound healing [18, 19]. AMG was another modern therapy that we combined with conventional treatment. AMG promotes the healing process by reducing scar tissue formation, reducing inflammation, having antibacterial properties, and providing a matrix for cellular proliferation [9]. Some recent studies have also shown that application of AMG is a safe and effective way to accelerate healing of chronic wounds [8, 20].

The combined use of MDT with other treatment strategies such as NPWT and AMG can be beneficial and effective in treating nonhealing necrotic wounds, especially in high-risk patients with underlying health issues such as diabetes, in the elderly, and others. Researchers recommend that medical teams and wound managers combine wound treatment strategies in order to promote the wound-healing process and subsequently decrease patients’ healthcare costs.

## Data Availability

Not applicable.

## Abbreviations

AMG: Amniotic membrane grafting
MDT: Maggot debridement therapy
NPWT: Negative-pressure wound therapy
